# Radon exposure and lung cancer: risk in nonsmokers among cohort studies

**DOI:** 10.1186/s40557-016-0099-y

**Published:** 2016-03-09

**Authors:** Sung-Soo Oh, Sangbaek Koh, Heetae Kang, Jonggu Lee

**Affiliations:** Department of Occupational and Environmental Medicine, Yonsei University Wonju Severance Christian Hospital, 20 ilsan-ro, Wonju, Gangwon 220-701 South Korea

## Abstract

Eleven cohorts of miners occupationally exposed to relatively high concentrations of radon showed a statistically significantly high risk of lung cancer, while three cohorts from the general population showed a relatively low concentration, but the results were not statistically significant. However, the risk of lung cancer tended to increase with increased radon exposure. The risk is likely to have been underestimated due to low statistical power. Therefore, additional well-designed studies on the risk of lung cancer in nonsmokers in the general population with relatively low concentrations of radon exposure are needed in the future.

In addition, country-specific preventive policies are needed in order to actively reduce radon exposure and lung cancer incidence in nonsmokers.

## Background

Radon is the second most common cause of lung cancer, and the most common cause in nonsmokers. Lung cancer has gradually been on the increase, especially in nonsmokers. To identify the cause of cancer in nonsmokers is very important because unlike some other types of cancer, early detection is difficult.

Epidemiological studies designed to assess human health risks from exposure to radon mainly consist of cohort mortality studies, residential case-control studies and ecological studies. The International Agency for Research on Cancer has declared radon to be a cause of lung cancer in humans, based on the results of experimental and epidemiological studies [1]. Compelling evidence of radon-induced health effects in humans derives from numerous studies of underground miners, particularly uranium miners exposed beginning in the middle part of the twentieth century in the United States and several European countries. Although these cohort mortality studies typically involved rather crude estimates of radon exposure levels in the working environment and inherent uncertainty due to confounding factors such as smoking status and coexposure to known or suspected human carcinogens (diesel exhaust, arsenic and silica dust), the results nevertheless consistently demonstrate increased risk of lung cancer with increasing exposure to radon in the working environment. These results are consistent across the various individual studies of mining cohorts and with analyses of pooled data from multiple cohorts.

In this paper, we reviewed the risk of lung cancer from radon for nonsmokers by examining occupational cohorts of miners and the general population among all the studies that are found through Pubmed.

## Review

### Studies of cohorts of radon-exposed miners

#### Yunnan, China [2]

In 1985, the Labor Protection Institute (LPI), the health research unit of the Yunnan Tin Corporation (YTC), the Cancer Institute of the Chinese Academy of Medical Sciences, located in Beijing and the U.S. National Cancer Institute initiated a historical cohort study of active and retired employees of the YTC. In 1976, the LPI conducted an occupational health survey among all active and retired employees who worked in its five major mining units. The survey covered almost 20,000 individuals of the approximately 44,000 current and retired YTC employees. The study population was defined as all workers who participated in the occupational survey and who worked at one of the five major mining units. The occupational survey was a census and therefore included all employees within these five units. Most YTC workers with some underground experience worked in one of the five units and were thus included in the study population (Table 1).

**Table 1:** Relative risk of lung cancer by smoking status and radon exposure [2]

	Cumulative radon exposure (WLM)				
	<100	100–199	200–399	400–799	> − 800
Nonsmokers					
Cases	2	2	1	16	4
Relative risk	1.0	2.2	0.7	9.9	9.2

Table excludes 4088 subjects (74 lung cancer cases) with unknown smoking data

*WLM* working level month

Among subjects with available data, 3 % of the lung cancer cases and 7 % of the noncases identified themselves as nonsmokers at the time of the survey. The RR of lung cancer increased with cumulative radon exposure (WLM) within nonsmokers. Smokers have a two to threefold excess risk of lung cancer compared to nonsmokers (data not shown).

#### West Bohemia, Czech Republic [3]

The study population was established by means of a search of the employment records of miners at the Jachymov and Horni Slavkov mines. A man was included in the cohort if he commenced underground work in the period 1948 to 1959; if underground work lasted at least 4 years; and if details of his employment history were available, including the type of work the man did and the specific mine shafts in which he worked, together with the relevant dates. A total of 4320 men satisfied these criteria and were included in the study. The men were followed until 1 January 1991 (Table 2).

**Table 2:** Lung cancer in miners according to smoking status [3]

Group followed	Mean WLM	Person-years at risk	Lung Cancer		Attributable cancers	Ratio of risk in smokers and nonsmokers
			Observed (95 % CI)	Exp.		
Nonsmokers	31.7	5138	3 (0.7–9.3)	0.3	16.6	1.6
Smokers	29.7	9420	19 (12.1–29.4)	11.7	26.1	
Total	30.4	14,558	22 (14.6–32.5)	12.0	22.6	

*WLM* working level month

Smoking history was obtained for only a portion of the miners (75 %). The observed lung cancer rate in the nonsmokers was compared with the expected cancer rate according to statistics on the male population in Czechoslovakia. The attributable annual lung cancer rate per 1 WLM in smokers was found to be 1.6 times higher than in nonsmokers. The ratio of the observed to expected lung cancer rate in nonsmokers was, however, considerably higher than in smokers.

#### Colorado Plateau, USA [4]

A cohort from the Colorado Plateau area of the United States consisted of 4137 men who worked in a uranium mine for at least one month and agreed to at least one health screening between January 1, 1950 and December 31, 1960. The cohort was followed until 1990. Additional follow-up through December 31, 2005, was obtained by linking workers to the National Death Index and the Social Security Administration’s mortality file by name, Social Security number and date of birth (Table 3).

**Table 3:** Standardized mortality ratios and standardized rate ratios by smoking status and cumulative radon progeny exposure category (lagged 5 years) for lung cancer among Colorado Plateau uranium miners, 1960–2005 [4]

	Cumulative exposure to radon progeny from uranium mining (WLM)				Trend slope, cases/WLM person-year
	<120	120– < 400	400– < 1000	> − 1000	
Never smoker					4.5 × 10^−6c^
No.	2	9	22	43	
SMR (95 % CI)	0.25 (0.03–0.89)	0.87 (0.40–1.7)	2.9 (1.8–4.4)	6.3 (4.6–8.5)	
SRR (95 % CI)	1.0 (ref)	3.5 (0.75–16)	13 (3.0–54)	29 (7.0–120)	

*SMR* standardized mortality ratio, *SRR* standardized rate ratio, *WLM* working level month

Nearly half (*n* = 34) of the lung cancer deaths among never-smokers occurred among American Indian miners. The standardized mortality ratio increased directly with WLM exposure level. The standardized rate ratios increased monotonically with WLM exposure for most never-smokers. Tests of trend were highly significant for never-smokers.

#### Ontario, Canada [5]

In this study, uranium miners were defined as men who either attended a chest clinic after 1954 and reported that they had worked for at least two weeks in a uranium mine in Ontario, or who were reported by a uranium mining company to have been exposed to short-lived radon progeny underground in a uranium mine in Ontario. In all 26,674 uranium miners including 1344 uranium mill workers were identified. 21,346 male uranium miners remained after exclusions.

About 20 % of the uranium miners in these surveys reported that they had never smoked. A logistic regression analysis of the smoking histories showed that an association between the proportion of uranium miners who never regularly smoked and the miner’s year of birth could be detected (*X*^2^ 24.2, *p* = 9 × 10^−7^). The results of the logistic regression analysis also indicated that the proportion of uranium miners who never regularly smoked increased 2 % for each 10-year increment in the year of birth (data not shown).

#### Newfoundland, Canada [6]

In a study of Newfoundland province of Canada, the cohort consisted of 1743 underground miners and 321 surface workers employed by either the St. Lawrence Fluorspar Company or Newfoundland Fluorspar Limited from 1950 to 1990 (Tables 4 and 5).

**Table 4:** Relative risk of lung cancer and fitted models, underground Newfoundland fluorspar miners, by cumulative radon progeny exposure and smoking status [6]

Smoking status		Cumulative radon progeny exposure (WLM)			
		<500	500– < 1500	1500– < 2500	2500+
Non-current	Deaths	6	7	4	2
	RR	1.0	4.80	5.17	5.22

The relative risk was adjusted for age and calendar period

*RR* relative risk, *WLM* working level month

**Table 5:** Excess risk of lung cancer, per WLM, by smoking status in underground fluorspar miners of known smoking status, Newfoundland, 1950–1990 [6]

Smoking status	Lung cancer deaths	ERR/WLM	95 % CI	*P*
Non-current	19	0.0025	0.0006–0.0093	0.03
Current	71	0.0055	0.0024–0.0168	
Overall	90	0.0046	0.0020–0.0144	

*ERR* excess relative risk, *WLM* working level month

Among nonsmokers, the RRs increased with increasing radon progeny exposure: 1.0, 4.8, 5.17 and 5.22. The ERR/WLM was 0.0025. This result was higher for current smokers than nonsmokers (*p* = 0.03). Attributable risk among never-smokers in this cohort was 0.65 ().

#### Malmberget, Sweden [7]

This study evaluated workers from the Luossovaara-Kiirunavaara Aktiebolaget (LKAB) mine in Malmberget, Sweden, located above the Arctic Circle. The miners were men born between 1880 and 1919 who were alive in 1930 and who worked underground in more than one calendar year between 1897 and 1976. Lung cancer mortality was investigated from 1951 to 1976 in 1415 Swedish iron miners (Table 6).

**Table 6:** Results of cohort studies of risk estimates of lung cancer in underground miners from exposure to radon progeny [7]

Cohort study	*N*	Mean years of follow-up after start of work	Absolute risk coefficient/106 person-yr/WLM	Relative risk coefficient/WLM
Sweden iron, 1951–76	1294	44	19	0.036
Smokers		44	22	0.024
Nonsmokers		44	16	0.107

*WLM* working level month

The relative risk coefficient for the nonsmokers was 0.107/WLM. This suggests that the dose required to double the risk of lung cancer is less than 10 WLM. Such a low value indicates that when a lag of 30 years is applied to calculations of effects of background doses of 0.1 WLM per year from radon indoors, a major proportion of the cases of lung cancer observed in nonsmokers among the general population may be accounted for by exposure to radon.

#### New Mexico, USA [8]

A cohort of 3469 males with at least 1 year of underground uranium mining experience in New Mexico was assembled and mortality followed through 31 December 1985. The cohort was assembled by matching lists of men who had undergone mining-related physical examinations at the Grants Clinic in Grants, NM, against company personnel records. From its opening in 1957, this clinic had performed most of the preemployment and follow-up examinations for the Grants area mines. Personnel records from the five principal companies operating in the 1970s and several smaller companies were reviewed to document that the subjects had worked underground in New Mexico uranium mines at least 1 year by 31 December 1976. This process selected 4044 men (Table 7).

**Table 7:** Relative risks for lung cancer by exposure to radon progeny, with and without adjustment for cigarette smoking, in a cohort of New Mexico underground uranium miners [8]

Exposure category	Cigarette smoking		
	Number of cases	Unadjusted (95 % CI)	Adjusted (95 % CI)
0–99.9	11	1.0	1.0
100–199.9	12	2.2 (1.0–5.1)	2.2 (0.9–5.0)
200–299.9	10	2.8 (1.2–6.7)	2.7 (1.1–6.6)
300–399.9	11	7.3 (3.1–17.2)	7.1 (3.0–16.8)
400–499.9	9	10.7 (4.3–26.4)	10.8 (4.4–26.7)
500–749.9	8	9.5 (3.7–24.2)	9.0 (3.6–23.0)
750–999.9	2	10.3 (2.2–46.9)	9.9 (2.2–45.5)
≥ 1000	4	12.3 (3.9–39.1)	13.5 (4.3–43.1)

This was calculated by Poisson regression with adjustment for ethnicity, calendar year and age

The relative risk for ever smokers compared with never smokers was 3.6 (95 % CI 1.3-10.0). The relative risk values changed little with adjustment for smoking. However, the deviance for the model including smoking was significantly less than for the model without smoking, indicating that smoking was an important risk factor.

#### Eldorado Beaverlodge, Canada [9]

The Beaverlodge uranium mine began operation in 1949, commenced full production in 1953, and closed in 1982. The cohort consisted of all male former employees who had worked at the mine since 1948, together with males currently employed at the mine at the termination of the follow-up period (31 December 1980). The cohort, so defined, consisted of 10,945 individuals, after excluding the Beaverlodge cohort consisting of 8487 individuals, 77.5 % of those originally defined as eligible.

This study did not collect data on smoking.

#### France [10]

Uranium mining began in France in 1946. The first mines, operated by the Commissariat a l’Energie Atomique (CEA), were in the Massif Central. The inclusion criteria are defined in terms of the period of first exposure and duration of exposure to radon and its decay products: this cohort includes all the uranium miners with a first experience of underground mining in the years 1946–1972 and with more than 2 years of underground mining.

This cohort was used to carry out a nested case-control study for lung cancer in order to examine the association of factors such as smoking.

#### Eldorado Port Radium, Canada [11]

The Port Radium uranium mine opened in 1930 and closed in 1960, with a brief closure between 1940 and 1942. The initial cohort consisted of all male workers employed at the mine since 1940 and who were known to be alive as of January 1, 1945. The final cohort study comprised 2103 workers employed between 1942 and 1960 at a uranium mine in the Northwest Territories, Canada.

This study did not collect data on smoking.

#### Radium Hill, Australia [12]

The Radium Hill mine was located in a remote area of eastern South Australia, and operated from 1952 to the end of 1961, producing uranium for export to Britain and the US. The mine was owned and operated by the South Australian Department of Mines. The study participants included 2574 persons employed at Radium Hill. Workers’ names, birthdates and job particulars (job type, starting date, stopping date) were abstracted from records kept by the South Australian Department of Mines. These records included only wage earners employed by the Department. The list did not include salaried employees (geologists, management and other professional staff) or contractors.

In this cohort, no risk for lung cancer was found among nonsmokers.

Radon risk models are examined here from epidemiological studies of radon-exposed miners. The lung cancer risk models from the miner studies were based on male workers occupationally exposed to radon. The Biological Effects of Ionising Radiation (BEIR) VI joint analysis of 11 cohort studies of radon-exposed miners (BEIR VI), the joint analysis of three European cohorts of uranium miners, in the Czech Republic, France and Germany (Table 8).

**Table 8:** Estimated excess lifetime risk of radon-induced lung cancer death (REID) in males and females up to age 75 years from age 30, based on a lifetime exposure constant at various radon concentrations using various risk models, assuming a multiplicative model for radon and smoking [16]

Lifetime risk of lung cancer death from radon exposure at home (%)							
Radon concentration (Bq/m^3^)	Exposure WLM/year	BEIR VI cohort miner model			European cohort miner model		
		Continuing smoker	Ex-smoker from age 50	Never-smoker	Continuing smoker	Ex-smoker from age 50	Never-smoker
Males							
20	0.09	0.70	0.32	0.04	0.46	0.22	0.03
50	0.22	1.70	0.79	0.11	1.12	0.53	0.08
80	0.35	2.68	1.25	0.17	1.77	0.84	0.12
100	0.44	3.36	1.57	0.21	2.22	1.06	0.15
200	0.88	6.55	3.11	0.42	4.37	2.11	0.30
400	1.76	12.5	6.10	0.85	8.45	4.15	0.60
600	2.64	17.9	8.97	1.27	12.30	6.14	0.90
Females							
20	0.09	0.56	0.28	0.05	0.36	0.19	0.04
50	0.22	1.36	0.69	0.13	0.89	0.46	0.09
80	0.35	2.16	1.09	0.21	1.40	0.73	0.14
100	0.44	2.70	1.37	0.27	1.76	0.91	0.18
200	0.88	5.31	2.71	0.53	3.48	1.82	0.36
400	1.76	10.30	5.34	1.06	6.81	3.59	0.73
600	2.64	14.93	7.88	1.58	10.00	5.33	1.08

*WLM* working level month

Table 8 shows estimates of REID for a European population. These calculations are based on exposure from age 30 up to 75 years to a constant radon concentration of 20, 50, 80, 200, 400 or 600 Bq/m^3^ using cohort models. Risks are presented separately for males and females, and for continuing smokers, never-smokers and persons who stopped smoking by age 50.

The lifetime risk of radon-induced lung cancer death by age 75 years for a male never-smoker, assuming that this individual has lived from age 30 years in a home with a radon concentration of 50 Bq/m^3^ (the European long-term average), is estimated to be in the range 0.08–0.11 %, according to the risk model used. These estimates rise to 0.30–0.42 % for male never-smokers in homes with a radon concentration of 200 Bq/m^3^ and to 0.90–1.27 % at 400 Bq/m^3^.

### Studies of residential radon cohorts

High-level occupational radon exposure is an established risk factor for lung cancer. Although a number of residential case-control studies have been published, few cohort studies have assessed the relationship between residential radon exposure and the development of lung cancer.

#### American Cancer Society Cohort [13]

Nearly 1.2 million Cancer Prevention Study II participants were recruited in 1982. Mean county level residential radon concentrations were linked to study participants according to zip code information at enrollment. Participants were at least 30 years of age and had at least one family member aged 45 years or more. A total 811,961 participants in 2754 counties were retained for analysis, among whom 3493 lung cancer deaths were observed until 1988 (Table 9).

**Table 9:** Adjusted HRs (95 % CIs) for lung cancer mortality per 100 Bq/m^3^ mean county-level residential radon concentrations (LBL) at enrollment (1982) stratified by selected risk factors, effect modification multiplicative scale, follow-up 1982–1988, CPS-II cohort, United States [13]

Characteristic	*n*	Lung cancer deaths	Fully adjusted HR (95 % CI)	*P*
Cigarette Smoking				
Never-Smoker	375,087	271	0.77 (0.47–1.25)	
Current	152,033	1792	1.20 (1.00–1.44)	
Former	203,253	941	1.09 (0.84–1.41)	0.66

Age, race, gender and state stratified and adjusted for education, marital status, BMI, BMI squared, cigarette smoking status, cigarettes per day, cigarettes per day squared, duration of smoking, duration of smoking squared, age started smoking, passive smoking, vegetable/fruit/fiber consumption, fat consumption, industrial exposure and occupation dirtiness index where appropriate.

No significant effect modification was observed by cigarette smoking status.

In the present study, a number of risk factors for lung cancer were less prevalent among participants. This would result in an underestimation of the association between radon and lung cancer risk.

#### Danish Cohort [14]

Between December 1993 and May 1997, 57,053 persons aged 50–64 years were enrolled in the prospective study “Diet, Cancer and Health”. The participants had to be born in Denmark, live in Copenhagen or Aarhus, and be cancer free at the time of inclusion. We followed each cohort member for cancer occurrence until 27 June 2006, identifying 589 lung cancer cases (Tables 10 and 11).

**Table 10:** Incidence rate ratio for lung cancer associated with the concentration of radon at the residence [14]

Radon (Bq/m^3^)	All residences, IRR (95 % CI)		
	Cases, *n*	Model 1	Model 2
Non-smoker			
< 17.6	27	1.00	1.00
17.6–39.5	24	0.91 (0.52–1.58)	1.16 (0.63–2.13)
39.5–66.1	22	0.78 (0.44–1.37)	1.25 (0.60–2.59)
> 66.1	26	0.85 (0.49–1.45)	1.48 (0.68–3.20)
Linear trend per 100 Bq/m^**3**^	99	0.82 (0.43–1.56)	1.67 (0.69–4.04)

Model 1 was adjusted for age by using it as the time scale in the Cox model

Model 2 was adjusted for age, sex, BMI, length of school attendance, socio-economic status, environmental tobacco smoke, fruit intake, alcohol intake, residence type, employment in an industry or job associated with higher risk for lung cancer, and traffic (time weighted average NOx exposure)

*IRR* incidence rate ratio

**Table 11:** Adjusted incidence rate ratios for lung cancer in association with a 100 Bq/m^3^ increase in domestic radon within strata of sex, NOx at the residential address, and ETS [14]

Interaction variable	Cases, *n*	Nonsmokers IRR (95 % CI)	*P*
Sex			
Men	46	1.35 (0.44–4.11)	0.52
Women	53	2.02 (0.70–5.76)	
NOx at front (ug/m^3^)			
< 21.8	48	1.80 (0.60–5.42)	0.92
≥ 21.8	51	1.68 (0.54–5.23)	
ETS			
No	34	1.19 (0.34–4.20)	0.45
Yes	65	1.99 (0.74–5.33)	

Adjusted for age, sex, BMI, length of school attendance, socio-economic status, environmental tobacco smoke, fruit intake, alcohol, residence type, employment in an industry or job associated with higher risk for lung cancer, and traffic (time weighted average NOx exposure)

*ETS* environmental tobacco smoke

Among nonsmokers, the incidence rate ratio was 1.67 (95 % CI: 0.69–4.04) and the incidence rate ratio was dose-dependently higher over the four radon exposure quartiles.

There was no evidence that the association between radon and risk of lung cancer was modified by sex, traffic-related air pollution or environmental tobacco smoke.

This cohort showed an insignificant association between radon and risk for lung cancer with an associated convincing dose-response pattern over the four quartiles of radon exposure. The lack of a significant linear response amongst nonsmokers was surprising, but only 99 nonsmokers developed lung cancer and power constraints may explain this.

#### Spain cohort [15]

Between 1992 and 1994, 241 randomly selected controls were enrolled in a population-based case-control study on residential radon and lung cancer by using 1991 census data for the Santiago de Compostela Health District. Initially, 500 persons from the general population were selected through sex-stratified random sampling. Of these, 391 met the eligibility criteria and 241 were finally included. Cohort follow-up ended on 31 May 2007.

In this cohort, no risk for lung cancer among nonsmokers was found.

## Conclusions

To date, cohort studies of miners with exposure at high concentrations of radon have shown increases the risk of lung cancer in nonsmokers. However, the risk is unclear at the relatively low concentration of radon exposure experienced by a few cohorts in residential settings. In the future, well-designed prospective cohort studies are needed.
